# Understanding the workplace needs of autistic adults in Singapore: insights to inform inclusive AI support

**DOI:** 10.3389/fpsyt.2026.1825357

**Published:** 2026-05-19

**Authors:** Delia DD Kan, Aricia Chuai, Karen Bong, Edwin Chng, MingWei Ang, Elinor Lim

**Affiliations:** 1 National Institute of Education, Nanyang Technological University, Singapore, Singapore; 2 School of Humanities, Nanyang Technological University, Singapore, Singapore; 3 Independent Researcher, Singapore, Singapore; 4 School of Psychology, Sport and Health Sciences, University of Portsmouth, Portsmouth, England

**Keywords:** artificial intelligence, autism, employment, inclusion, large language models, workplace

## Abstract

**Introduction:**

Autistic adults face persistent challenges in obtaining and sustaining meaningful employment. Despite growing attention to workplace inclusion, research on autistic adults’ employment experiences in non-Western contexts remains scarce. The potential of emerging technologies, such as large language models (LLMs), to support workplace integration is also still largely unexplored, yet off-the-shelf models may reflect neurotypical norms that risk reinforcing masking or overlooking neurodivergent needs. This qualitative, participatory study therefore centered autistic adults’ perspectives to understand workplace experiences and identify potential LLM affordances that could facilitate integration.

**Methods:**

Twenty autistic adults with at least 3 months of work experience were recruited in Singapore. Data was collected through semi-structured group discussions. Using reflexive thematic analysis, we identified workplace challenges, needs, and potential support LLMs can provide. We had a multidisciplinary team of autistic and non-autistic researchers, and autistic perspectives actively shaped the design, conduct, and interpretation of the research.

**Results:**

Two overarching themes emerged: (1) assumed neurotypicality of the workplace, evident in work processes and social participation, and (2) need for workplace inclusivity, supported through both individual accommodations and systemic change, including identity-affirming support, alignment of work design and tools with neurodivergent working styles, empowered access to supports and accommodations, and shared responsibility for workplace integration. Potential LLM functionalities involve supporting executive functioning, encouraging self-reflection, and fostering mutual understanding between autistic employees and their coworkers.

**Discussion:**

Workplace barriers for autistic employees often stem from assumed neurotypical norms rather than individual deficits. Participants reported challenges related to ambiguous work processes, implicit social expectations, and executive functioning demands, which reflect a mismatch between workplace structures and neurodivergent ways of working. Crucially, inclusivity cannot rely solely on individual accommodations; meaningful workplace inclusion requires systemic change. Designing LLM tools that align with neurodivergent working styles can complement systemic inclusivity efforts and empower autistic employees. Implications and future directions are discussed.

## 1 Introduction

Autism is a neurodevelopmental condition marked by differences in social communication and interaction, alongside restricted and repetitive patterns of behavior, interests, or activities (1). Increasingly, autism is understood through the neurodiversity paradigm, which recognizes cognitive variation as a natural part of human diversity and calls for differences to be respected and accommodated rather than pathologized (2). Individuals on the autism spectrum are also recognized as highly heterogeneous, with diverse strengths, preferences, and support needs that cannot be addressed through a one-size-fits-all approach (3). Worldwide, the prevalence of autism has been increasing (4), a trend commonly attributed to increased recognition and evolving diagnostic practices (5, 6). Similar patterns are found in Singapore. Although no nationally representative prevalence study has been conducted to date (7), there has been growing demand for autism-related supports, including the expansion of special education provision for students on the autism spectrum over the past decade (8, 9). As more autistic youth transition to adulthood, attention has shifted toward adult life outcomes, particularly employment, which plays a critical role in shaping well-being and community participation.

### 1.1 Barriers to sustained employment

In spite of many strengths autistic individuals bring to the workplace (e.g., strong memory and attention to detail; 10), access to employment and job retention for autistic adults remain key issues internationally, with many underrepresented in the labor market (11–13). Autistic adults often show lower workforce participation than non-autistics and those with other disabilities (14). High rates of underemployment are also common, with many autistic adults occupying part-time, casual, or lower-skilled roles that do not match their skills or fully utilize their capabilities (11, 14). Although no Singapore-specific employment data on autistic adults are available, figures for people with disabilities indicate substantially lower workforce participation than the general population, with 33.6% employed in 2024 compared with 68.2% of all residents, suggesting similar patterns locally (15). These disparities are concerning, as unemployment and underemployment have been associated with poorer mental health outcomes among individuals with disabilities, particularly autistic individuals. Specifically, they have been linked to elevated levels of stress, anxiety, and depression, as well as reduced self-esteem and life satisfaction (16, 17).

Low workforce participation rates could be attributed to hurdles encountered at multiple stages of the employment journey. From the onset, traditional interview processes may inadvertently disadvantage autistic job seekers. While autism presents uniquely in each person, challenges related to social communication and interpersonal functioning are frequently cited as key barriers to employment (18). These challenges can be intensified by the neurotypical expectations embedded in standard interview formats, which often emphasize conventional social practices such as rapid conversational exchanges and eye contact (19, 20).

After attaining employment, autistic adults may also experience difficulties integrating into the workplace. Some studies have pointed to factors such as sensory sensitivities and a strong need for routine and consistency, as contributing to difficulties in navigating work environments that are not designed to accommodate these needs (21, 22). Beyond these structural challenges, social stigma may contribute to social exclusion and, in turn, the adoption of coping strategies, such as camouflaging or masking, to hide autistic traits. Such strategies may be linked to longer-term strain and burnout, with adverse implications for psychological well-being (23). Nonetheless, existing research remains largely focused on recruitment and job attainment (24), leaving a gap in understanding what happens after employment commences. Autistic adults themselves have highlighted the need for more studies addressing workplace integration and retention (11). Addressing this gap is therefore critical for informing supports and practices that enable sustained employment.

### 1.2 Cultural and workplace contexts

Autistic employees’ workplace experiences may also be shaped by broader cultural contexts. Autism-related knowledge, stigma, and stereotypes vary across societies, influencing how individuals on the spectrum are perceived and supported. For instance, Cheng et al. (25) reported lower levels of autism knowledge and higher explicit stigma in Hong Kong compared with the U.S. and the U.K.

One widely studied dimension of national culture is individualism-collectivism (26). From this perspective, individualist cultures (e.g., the U.S., Canada, Australia) tend to emphasize personal autonomy, whereas collectivist cultures (e.g., Malaysia, South Korea, Singapore) tend to prioritize group cohesion and loyalty (26, 27). Differences in social communication and interaction, which are core diagnostic features of autism, can make job integration more challenging for individuals on the autism spectrum, as success depends not only on technical skills but also on an understanding of workplace norms and socio-cultural expectations (28). These demands may be especially pronounced in collectivist contexts. For instance, in many Asian workplaces, effective performance often involves attending to subtle social cues and understanding implicit expectations around collaboration and hierarchy (29). In such group norms where harmony and conformity are highly valued, deviations from the norm may stand out and be judged more harshly, increasing pressure to follow unwritten rules around hierarchy and social interactions (30).

Even so, we contend that cultural dimensions such as individualism-collectivism may risk oversimplifying diverse lived experiences. There is a need for more in-depth explorations of what these dynamics actually mean in practice and specific contexts. Singapore remains inadequately represented in autism employment literature, and much of the existing neurodiversity research and advocacy has been conducted in Western, English-speaking contexts, reflecting cultural, linguistic, and historical assumptions that may not fully translate elsewhere (2). To the best of our knowledge, autistic adults’ employment experiences in Singapore remain largely unexamined in the empirical literature. Local work to date appears limited to an isolated case-based report (31), underscoring the need for culturally grounded research that centers autistic adults’ perspectives.

### 1.3 User-centered supports and technology

Support strategies such as job coaching provide individualized guidance to help employees navigate both formal requirements and informal workplace expectations (32). Job coaching is highly valued by both autistic employees and their employers and is positively associated with improved workplace integration and retention (33). However, such support is costly, resource-intensive, and often in high demand, limiting its accessibility (32). In Singapore, this constraint is further compounded by the limited number of trained job coaches, with only about 200 professionals available across social service agencies, schools, and employment support organizations in 2024, compared with tens of thousands of people with disabilities (34).

In response, technology has been increasingly explored as a complement or alternative to human coaching to support autistic individuals in employment. Notably, virtual reality (VR) technologies have been used to simulate workplace scenarios for training, including teamwork and job interviews (35, 36). Recent advancements in artificial intelligence (AI), particularly large language models (LLMs), have also shown promise in making information more accessible to users and providing scalable, low-stress support, including conversational simulations and scenario-based guidance (37, 38). Some work has also explored combining VR with LLM-powered avatars to support role-playing and soft skills training for autistic individuals in vocational training programs (39). Other AI-based interventions have also been shown to support autistic adults in developing communication skills and emotion-regulation skills to cope with anxiety (40, 41), while also helping non-autistic individuals build understanding of neurodiversity and more inclusive practices (42). Together, these combined supports have the potential to improve psychosocial outcomes for autistic adults, including greater workplace integration and enhanced self-esteem, contributing to overall vocational success. However, it should be noted that several AI interventions have also focused on training autistic individuals to display more neurotypical traits, such as maintaining eye contact (43). While intended to support social functioning, such approaches may instead reinforce masking and potentially worsen psychosocial outcomes.

Most LLM tools remain early-stage pilots or proof-of-concept projects, and their application in employment contexts is still limited. Furthermore, concerns about neurotypical bias in LLM training data suggest that off-the-shelf models may encourage masking, overlook neurodivergent communication styles, and leave autistic users unprepared for complex social interactions (44, 45). This points to a need for more autistic input when designing and implementing technology tools.

Although technology-based employment supports have been studied in Western countries and parts of East Asia, they have not been extensively reported in Southeast Asian contexts such as Singapore, particularly regarding LLM applications (Chuai et al., under review). Coupled with the abovementioned ethical concerns surrounding mainstream LLMs, it remains unclear to what extent these tools can meet the needs of autistic users in Singaporean workplaces.

### 1.4 Community-inclusive design and participatory approach

To understand how LLMs might support autistic adults in Singaporean workplaces, it is crucial to first center lived experiences of the local autistic community. Without input from autistic individuals themselves, research risks reinforcing neurotypical norms and failing to meet users’ needs. Participatory research emphasizes the meaningful involvement of autistic individuals and their allies in decisions about what research is conducted, how it is implemented, and how outcomes are evaluated (46, 47). Studies suggest that research incorporating the perspectives of autistic adults yields insights that are more relevant, culturally sensitive, and reflective of real-world needs (48, 49). However, although there have been increasing calls for community engagement in autism research, truly participatory approaches remain uncommon and, in some cases, are implemented only superficially (50).

### 1.5 Study aim and research questions

To address this gap, this participatory qualitative study aims to identify the workplace needs and challenges faced by autistic adults in Singapore and explore how LLMs might support them. To frame this inquiry, we draw on the concept of technological affordances. Affordances refer to the possibilities for action that technologies offer users through the interaction between their technical capabilities and users’ perceptions of how they can be used (51).

While one longer-term goal is to inform the development of inclusive technologies, the core focus of the present study lies in understanding what needs to be in place to allow autistic individuals to thrive and sustain at work. Guided by a participatory ethos, this study centers autistic adults’ priorities and lived experiences in the hopes of gaining more insight into the supports and conditions that could enable sustained employment, reflecting the vision of Singapore’s Enabling Masterplan 2030 (52). Specifically, the study addresses the following research questions:

RQ1: What workplace needs and challenges do autistic adults in Singapore encounter?
RQ2: What potential affordances of LLMs do autistic adults desire in navigating workplace challenges and needs?

## 2 Methods

### 2.1 Positionality statement

The research team included both autistic and non-autistic researchers. Our approach was grounded in an autism-affirming, participatory orientation, with the aim of centering autistic expertise throughout the study. One autistic researcher co-led the design of the workshop activities, facilitated group discussions, and contributed to shaping the participatory approach, supporting the accessibility and relevance of the sessions to autistic participants. The non-autistic researchers contributed experience in participatory methods and expertise in developing the LLM chatbot prototype. All but one researcher was based in Singapore during the course of the project, and the team’s professional experience related to disability, education, and employment, was largely situated within the Singapore context. At the same time, team members brought diverse educational backgrounds and training from Singapore, the U.S., and the U.K., in the fields of education, sociology, and psychology, which shaped the perspectives and assumptions we brought to data collection and interpretation.

### 2.2 Study design

This study used a participatory, qualitative design, within which workshops were conducted with small groups of autistic adults. Each workshop session consisted of (1) a semi-structured focus group discussion exploring autistic adults’ workplace needs and their perceived and desired affordances of large language models, followed by (2) hands-on interaction with an autism-affirming chatbot prototype and a brief post-use reflection. Multiple data sources were collected across the session; however, the present manuscript reports analyses from the focus group discussion component only. Findings from the prototype interaction and post-use reflection will be reported separately so that each component can be discussed in sufficient depth, rather than presented superficially within a single manuscript.

### 2.3 Participants

We recruited participants through multiple avenues, including outreach on social media, engagement with higher education institutions, and community organizations that provide employment support for neurodivergent individuals. Eligibility criteria required participants to be autistic, aged 21 years or above, and to have at least three months of work experience. Both formally diagnosed and self-identified autistic adults were eligible, recognizing barriers to obtaining a diagnosis and aligning with inclusive autism research practices (53). The study received approval from Nanyang Technological University’s Institutional Review Board (IRB-2025-105), and written informed consent was obtained from all participants. The study was also preregistered on Open Science Framework (OSF; https://osf.io/fstw7). The final research questions reflect a refinement of the preregistered questions, with no changes to recruitment or data collection procedures.

A total of 20 autistic adults in Singapore were recruited for this study. Participants had a mean age of 29.2 years (SD = 7.51; range: 21–46), and were predominantly male (n = 14, 70%). Regarding autistic identity, 75% (n=15) reported a formal clinical diagnosis, while 25% (n=5) were self-identified. The sample was highly educated, with the majority (45%) holding a university degree. Half of the participants (50.0%) were employed full-time, six (30.0%) were currently attending university, three (15.0%) were seeking employment, and one (5.0%) was self-employed. In terms of working experience, participants reported a mean of 5.87 years (SD = 7.00), with a range of 6 months to 21 years. The most commonly reported sectors were Office/Admin (n = 13), Tech/IT (n = 7), Hospitality, Retail/Sales, and Education (n = 6 respectively), with participants often reporting experience across multiple sectors. Demographic and employment breakdown for study participants can be found in **Table 1**.

**Table 1:** Participants' demographics and employment details.

Pseudonym	Age	Gender^1^	Ethnicity	Field(s) of work	Employment status and mode^2^
Alex	34	M	Chinese	Office/Admin; Education; Hospitality; Health & Social Services; Tech/IT; Creative/Media	Full time, Hybrid
Ben	28	M	Chinese	Office/Admin; Education; Health & Social services; Public Service	Full time, Hybrid
Casey	42	F	Chinese	Office/Admin Education; Retail / Sales; Tech / IT; Logistics; Manufacturing	Full time, Hybrid
Deborah	33	F	Chinese	Office/Admin; Education; Health & Social Services	Full time, Fully on site
Elliott	24	M	Chinese	Office/Admin; Tech/ IT	Full time, Fully on site
Freddy	34	M	Chinese	Office/Admin; Tech / IT	Seeking employment
Gary	29	M	Chinese	Tech/IT	Seeking employment
Henry	31	M	Chinese	Office/Admin; Education; Hospitality; Logistics; Facilities	Full time, Fully on site
Isaac	26	M	Chinese	Office/Admin; Retail / Sales; Tech/ IT	Seeking employment
Jackson	22	M	Chinese	Hospitality; Events	Full time, Fully on site
Josephine	46	F	Chinese	Office/Admin	Full time, Not reported
Lyon	23	M	Chinese	Office/Admin	Full time, Hybrid
Marcus	25	M	Chinese	Office/Admin; Lab/Scientific Services	Student
Ning	44	F	Chinese	Education; Creative/Media	Self-employed
Oliver	24	M	Chinese	Creative/Media; Research	Student
Patrick	25	M	Chinese	Office/Admin; Retail / Sales	Student
Nathan	25	M	Malay	Hospitality; Retail / Sales; Tech/ IT; Logistics	Full time, Fully on site
Rachel	24	F	Chinese	Hospitality; Retail / Sales; Logistics; Public service	Student
Sam	21	NB	Chinese	Hospitality; Retail / Sales	Student
Tim	23	M	Chinese	Office/Admin; Manufacturing; Public Service	Student

**^1^**M, Male; F, Female; NB, Non-binary/genderqueer, **^2^**At time of study data collection.

### 2.4 Procedures

Five workshops were conducted between August and October 2025, each lasting approximately 2.5 hours, with each participant attending one session only. The first workshop had six participants; following this session, we found that smaller groups were more conducive to deeper discussion and engagement, and all subsequent workshops were conducted with three to four participants. Sessions followed a structured schedule with fixed time allocations for each component. We acknowledge that the larger group size in the first session may have limited opportunities for individual sharing relative to subsequent sessions.

At the start of each session, the PI/co-PI introduced the study background, and participants were informed that they could step away or take breaks when needed. This was followed by brief icebreaker introductions before starting the focus group discussions. Discussions followed a semi-structured format, with the first half focused on participants’ workplace experiences and the second on imagined LLM functionalities. The discussions were facilitated mainly by the team’s autistic researcher, and was supported with accessibility scaffolds, including real-time visual summarization of key points on a board written by a research team member, as well as printed discussion prompts that participants had filled out prior to the discussion and could annotate and refer to during the discussion. After the discussions, participants engaged with a hands-on interaction with an autism-affirming chatbot prototype and a brief post-use reflection. Each participant was provided with SGD$50 upon completing the workshop session.

### 2.5 Data analysis

All discussions were audio-recorded and transcribed verbatim using MAXQDA’s transcription tool, with transcripts subsequently reviewed and corrected through manual checking to ensure accuracy. For the present paper, only the transcripts from the group discussions were included in our analysis. Before commencing analysis, we conducted summary verification. Participants were provided with a summary of the key points they had shared during the workshop and invited to confirm factual accuracy and intended meaning within 2 months of attendance. Due to an administrative oversight, three participants from the final workshop were not contacted within this timeframe and were therefore not included in this process. Of the 17 participants who were contacted, 7 responded, and none requested any corrections to the summary of their contributions.

This study employed reflexive thematic analysis (RTA; 54) to address the two research questions. We chose RTA because it provides a flexible, interpretive approach for identifying patterns of meaning across participants’ accounts of workplace experiences, while recognizing the active role of researchers in knowledge production. We took a primarily inductive approach, attending to both semantic (explicit) meanings and latent (underlying) patterns across participants’ accounts. Analysis followed the six phases of RTA outlined by Braun and Clarke: familiarization, coding, generating initial themes, reviewing and developing themes, defining and naming themes, and writing up.

Following familiarization, the first two authors independently generated initial codes for the main portion of the transcript related to participants’ workplace needs. After coding transcripts from each workshop, the coders met to discuss emerging patterns, clarify and refine interpretations, and reflect on similarities and differences in their perspectives. Consistent with a reflexive approach, these discussions were used to deepen engagement with the data rather than to achieve coder agreement. Intercoder reliability was not calculated, as this is inconsistent with the reflexive approach adopted, in which divergent readings are treated as analytic resources rather than discrepancies to be resolved. Once all workshops had been coded, we moved to theme development by re-engaging with the dataset as a whole and asking what broader patterns of meaning were evident across workshops. Candidate themes were iteratively developed and refined through repeated engagement with the dataset and team reflexive discussions, with attention to coherence within themes, distinction between themes, and relevance to the research questions. After the themes related to workplace needs have been developed by the team, the second author independently analyzed transcript portions related to desired LLM affordances, identifying functionalities and desired features proposed by participants that are related to the workplace needs identified. This was subsequently reviewed by the first author.

## 3 Results

**Figure 1:**
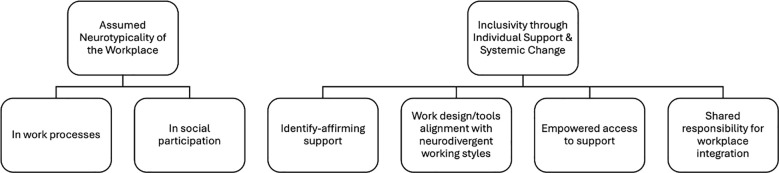
Summary of themes.

We identified two overarching themes in participants’ workplace experiences: (1) the assumed neurotypicality of the workplace, and (2) inclusivity through individual support and systemic change. The first theme encapsulates how workplace norms often assume neurotypical ways of working and communicating, while the second shows that meaningful inclusion can only occur when individual-level support is in place and systemic organizational practices evolve in tandem. **Figure 1** outlines the two themes and six subthemes, which will be discussed in turn. We refer to participants using pseudonyms and gender-neutral pronouns (singular “they/them”) throughout, as pronoun preferences were not collected.

### 3.1 Assumed neurotypicality of the workplace

This theme captures how participants experienced the workplace as structured around implicit neurotypical norms. Rather than being explicitly defined, procedures and standards of performance were often assumed to be intuitively understood. This section is organized into two sections: work processes and social contexts. In work processes, employees were expected to infer unstated steps which could be misaligned, leading to misunderstandings. They are also expected to adapt rapidly to shifting demands at work and manage ambiguity in tasks and processes without structured guidance. In social contexts, participation was similarly shaped by assumptions about societally normative communication styles, conversational interests, and acceptable forms of sociability. More details are provided below.

#### 3.1.1 In work processes

##### 3.1.1.1 Managing ambiguity and uncertainty

###### 3.1.1.1.1 Ambiguity in work processes and outcomes

Participants described workplaces as environments with limited clarity, where roles and tasks are governed by unspoken, neurotypical expectations. These expectations encompassed both what counts as successful performance (the desired outcome) and how work is expected to be carried out, including the often-implicit rationale for processes. Workplaces often operated on the assumption that employees would intuitively understand procedures and role demands without these being explicitly articulated: “They expect you to already know certain things even though you don’t fully know how they operate” (Gary). Instructions were frequently delivered as broad directives, requiring participants to infer performance expectations based on their own prior knowledge and assumptions, which did not always align with what supervisors intended. As Ben shared: “The supervisor might have a lot of presumptions that you know what to do but for many of us, we cannot read below the iceberg”. The use of slang, abbreviations, and acronyms by colleagues also increased the need to interpret and decipher implicit meanings. This ambiguity was described across industries, including hospitality and event work, where training and guidance could be similarly implicit: “Then they’ll be like, ‘Oh, you put the plate down, then you fold the napkin’… It’s a very hands-off approach” (Sam). As a result, participants often had to decide whether to make judgement calls of expectations for work assignments independently or to seek clarification to accurately meet expectations.

However, clarification seeking on work tasks was complicated by also having to meet implicit hierarchy expectations and manage neurotypical communication norms shaping both whether participants felt able to seek clarification and how they were expected to do so. Gary described a felt “distance” in interactions with supervisors, which made it difficult to ask for help even when clarification was necessary to complete tasks accurately. Marcus highlighted that even when they did seek support, they had to account for their supervisor’s preferences and unspoken norms around chain of command. Reflecting on their time in Singapore’s compulsory military service, Marcus shared: “So, by habit I just go with NSF (National Serviceman Fulltime; work seniors) because it’s like closer in age, seems more approachable compared to my actual boss. So there were issues with this lah then my boss end up insisting that because she’s in the chain of command, I should ask her instead.” (“Lah” is a Singapore English (Singlish) expression that softens or adds emphasis depending on context. It has been preserved in this manuscript as part of participants’ natural speech). Together, participants’ accounts suggest that clarification involves knowing who to approach, how to ask, and how to navigate the context appropriately.

Participants also described risks of being perceived as rude or demanding when seeking clarification due to differences in pragmatic norms between autistics and non-autistics. Ben reflected that being direct, while efficient from their perspective, could be read as demanding: “We will just say, ‘Can you help me with this task? I don’t know how to do, ’ but then … the other people see is… ‘Wah, you suddenly come to me and demand me to help you, I got my own things to do.’” They described how small, often unspoken expectations (e.g., checking availability first) could shape whether a request was received as appropriate, yet these expectations were rarely made explicit. When misunderstandings occurred, Ben shared that colleagues often did not provide direct feedback, instead “keep it to themselves … gossip, ” which “leads to breakdown in trust.” This dynamic created a self-reinforcing cycle, where earlier negative interactions made it even harder to seek clarification or feedback from colleagues. It also impacted social participation, with colleagues ostracizing the autistic employee, and in some cases leading to formal repercussions: “Then you go report me to the supervisor then it cause me to lose the job” (Ben).

Being able to seek clarification also depended on the availability of accessible supervisory support and clear help-seeking pathways. Participants noted that supervisors might not have time to provide detailed guidance, even when greater explicitness would be helpful. Others described that competitive work cultures could negatively impact knowledge sharing. In a commission-based role, Sam explained that competition for earnings meant colleagues were “less inclined to … really teach you, ” and they felt “it was a lot of like, you need to figure out how to sell things yourself.” Together, these accounts suggest that managing work assignments is not only a matter of interpreting unclear instructions. Even when participants took initiative to seek clarification, they still had to navigate workplace expectations shaped by hierarchy, competition, and time pressure, where they are expected to know how to respond and seek support appropriately, even without explicit instructions.

###### 3.1.1.1.2 Ambiguity in managing a workday

Beyond managing unclear work tasks, participants described a second layer of ambiguity in how to execute work in environments that assumed employees could independently generate structure. Participants were expected to continuously self-structure across the task lifecycle, including planning and sequencing activities, prioritizing what to start with, and recovering from missteps, often without explicit scaffolding.

To cope with these demands under uncertainty, multiple participants described relying on “anchors” that externalized structure and reduced the cognitive load of initiating, sustaining, and recovering within task sequences. Anchors took multiple forms, including temporal anchors (stable routines, time blocks, and deadlines), procedural anchors (standard operating procedures and step-by-step workflows), informational anchors (curated options e.g., for lunch break), and environmental anchors (organized physical systems that make materials and processes easy to locate and communicate). Although anchors helped participants make work more manageable, they were often effortful to develop and could break down when contexts shifted. Casey described how tasks could feel overwhelming until they created their own tracking structures, noting that “we are not good at building structures”. Participants also shared that when a step went wrong, or when a familiar procedure did not apply in a new context, they needed structured support to recover the process. Marcus shared how “if I get a step wrong, then they mess up the whole sequence, ” and the effort required to “find my bearings … where did I last get correct, ” rather than “go all the way back [to] the beginning, ” alongside the cascade of doubt that led them to “confirm check, every detail” and proceed very slowly. This was especially destabilizing when a previously learned step was invalidated due to changes in context and application: Marcus further described being told a step was wrong and then “question[ing] all the previous steps and all the subsequent steps, ” such that “I can’t use that as my anchor anymore … I’m just drifting around.”.

To make task expectations more explicit and accessible, participants highlighted the need for paced and structured information delivery, which they saw as a gap that an LLM tool can close. Participants desired LLM functionalities were aimed at simplifying and clarifying complex or fast-changing information, such as breaking down abstract instructions, emails, or verbal requests into step-by-step guidance delivered at a manageable pace. As Josephine explained: “Sometimes the technical information given or written is too complex and confusing because it’s too long. It’s better to rephrase it into clearer and simpler instructions.” Participants also shared how they hoped LLMs could support the development of certain anchors, for example, procedural and temporal anchors. As Rachel said: “I was thinking it can make, like, a task list or checklist of things I need to do for the day … like help me to break down tasks into smaller segments and which one to prioritise first, ya.”, suggesting her hope for the LLM to support the creation of workflows, and proposal of deadlines.

Participants also suggested the need for workflow-oriented support, including task sequencing, progress tracking, and decision-making under uncertainty. Relatedly, Marcus described a desired LLM functionality that could meet this need: “[Able to] list all possible permutations of a given procedure. Variations will be highlighted compared to other steps, so that we know what other steps would stay the same, [what would] change, and where exactly [these variations are].”.

##### 3.1.1.2 Dealing with real-time demands and managing changes

Participants highlighted that workplaces often treat the ability to adapt to and manage change as a baseline competency, with little recognition of the cognitive and emotional effort that adaptation can require for autistic employees. They described changes at two timescales. The first involves real-time, rapid shifts that require immediate reorientation, both in task demands and in communication. The expectation to rapidly reprioritize, switch tasks, and respond “on the spot” (Gary) can intensify cognitive load, particularly when the response must also be socially calibrated. This includes having to answer within an implicitly acceptable timeframe, maintain coherence, and navigate group discussions, such as in team meetings, where conversational turns move quickly. Participants described demands in real-time group conversations as multi-dimensional: sustaining attention to follow what others are saying, organizing and finding the right words to verbalize coherent response, and simultaneously monitoring the social flow to determine when it would be appropriate to enter the conversation when there are no explicit cues to contribute. As Alex shared: “I mean, it’s either I pay attention and then I can’t really think of what to say, or I don’t. And then I already know what I want to say, but then it’s not connected to whatever people are saying.” These interactional demands also become salient in high-stakes moments, such as client-facing or interpersonal conflict situations, where employees are expected to regulate their own emotions, find the “right” words, and de-escalate tensions in real-time.

The second form of change occurs over time, through shifts in procedures and routines that require updating an established internal script. Participants noted that variations in task procedures, even when minor, can be difficult when work is organized around standardized routines and how new steps fit within an existing sequence is not explicit. Routine changes also extend beyond the task itself to the broader ecology of work, such as rotating between venues, moving between workplaces, or adjusting to shift schedules (day and night shifts). As Lyon shared when they had to change jobs: “What if, after months of settling into a routine, you then suddenly have to uproot it because you’ve got a new job … imagine you, you spend all that time trying to remember every single little bit here and there, and then you go up and move to a new area. And then suddenly you have to start all over again.”.

#### 3.1.2 In social participation

Beyond task structures, participants noted that social integration in workgroups is generally shaped by neurotypical expectations about how people are supposed to engage in informal interactions, such as spending lunch time together, chatting about topics of interests unrelated to work. This aligned well with some of the participants’ desires for social participation at work. Gary described a desire to build more genuine friendships: “How to form a more genuine bond or connections instead of just work stuff. Can talk about life together.” However, this desire did not translate into their everyday experience of workplace interaction, which remained largely confined to task-related communication. They added: “Sometimes they (co-workers) only talk to you about work-related stuff, to others they might talk about more things. A lot of times, it just feels like you are there physically, but you’re not really there. You’re just there to do work. You’re just there as a placeholder. You can just be there listening and stuff like that, then you are at times just invisible” (Gary).

Building on earlier struggles of real-time conversational demands in work tasks and meetings, similar pressures also shaped informal social participation at work. Gary described real-time social interactions as highly unpredictable and context-dependent: “You can’t prepare in advance. So every time on the spot, people talk to you, you need to think of how to reply. If it’s a normal day-to-day conversation, it’s mainly unpredictable. You have no idea what will come up.” Participants also described how their narrow interests and age gaps could also limit their participation in informal workplace interactions. Patrick noted that their personal interests did not align with those of their colleagues, which made casual conversation feel effortful rather than natural: “Most of the time I have to figure out what my colleagues are talking about ‘cause … They may have normal interests … They do stuff that I just don’t find interesting … I’ll try my best to participate just to show that I do care. Just that it’s stuff that I can’t really get myself into.”.

In terms of potential support from LLMs, Tim highlighted the desire for LLMs to support such dynamic interactions, noting that responses vary by individual and daily mood: “I would like the LLM to assist me more with this … When it comes to people interaction, there’s no one-size-fits-all answer. It depends on who you’re dealing with. And people have different moods every day. If you say one thing to the same person on a different day, the response might be different.” Participants also noted that current LLMs may be limited in this aspect, as the modality of the support needs to allow for real-time assistance. Conversations tend to move too quickly for tools that require users to pause, reconstruct what was said, and type in a query before receiving guidance.

While some participants desired building friendships, other participants emphasized the importance of prioritizing personal regulation during the workday, such as seeking quiet or time alone. Josephine described choosing to spend lunchtime alone, while noting that this preference was often misinterpreted by others: “During lunchtime, just be alone, sit in the office or sit somewhere quiet, every day like that is okay. But to some people, they might find you strange or weird or antisocial. They will have difficulty understanding why— they will think that you are antisocial.” Similarly, Oliver described mandatory social activities (such as team-bonding events) as strange to navigate and unnecessary for work, indicating different expectations towards activities that have no clear relation to their job role: “Maybe I’m just inflexible or have a certain idea of the workplace? I don’t see such things as necessary. I want to be detached or impersonal at work and not be forced to participate in such activities. So it’s hard to manage expectations.”.

However, over time, such relational incongruence may accumulate, leading to misunderstandings and social exclusion, as described by Ben: “I think exclusion at the workplace is quite common. If you are working in a neurotypical majority workplaces, which most workplaces are, and then if people find that you are not able to fit in with the group, then they will start to exclude you from lunch or exclude you from other activities and over time this gap widens … and then unfortunately it could lead to the autistic person resigning or feeling … socially left out in the workplace.” Thus, effective integration depends on workplaces recognizing and accepting these self-defined belonging goals rather than enforcing a single standard.

### 3.2 Inclusivity through individual support and systemic change

This theme captures participants’ views that inclusion depends on both individual self-awareness and skills, and supportive workplace structures. At the individual level, employees need to understand their own strengths, challenges, and support needs. Without this self-knowledge, even well-designed systemic initiatives may be limited in effectiveness. However, clarity about one’s needs is only part of the picture. Macro-level factors, including organizational structures and workplace culture, may also impede inclusion if they are structurally rigid. Social inclusion initiatives are most effective when employees have access to meaningful support and are situated within systems that actively reinforce inclusion, fostering participation through a reciprocal relationship between individual understanding and workplace culture.

#### 3.2.1 Need for identity-affirming support

Latent interpretations of participants’ accounts suggested a need for identity affirming support that helps individuals make sense of their needs and relate to themselves with greater dignity in workplaces structured around neurotypical norms. Two issues emerged around support needs. First, participants conveyed difficulty identifying and naming their own support needs even before considering whether, when, or to whom to disclose them. They also shared that this has implications for the usefulness of LLM-based tools as supports, because currently such systems can only respond to what users are able to notice, recognize, and include in their prompts. As Isaac explained: “I think that some people, especially with ASD may have a lack of self-awareness in that they may not know what symptoms they have. They may know they have ASD. But unless someone points [something] out … You may not be aware or cognizant of it. And that missing piece will not be put into your [LLM] prompt.”.

Second, even when a need was recognized, participants described uncertainty about how to translate it into accessible and relatable language that others can understand and act on, particularly in contexts where neurodivergence remains relatively unfamiliar and support practices are not well established. In these situations, the autistic employee is often expected to explain what support they need, and they feel that they have to do it in a way that still makes them seem capable, rather than be seen as demanding or overly reliant on help. As Lyon shared: “One of the larger challenges of being a neurodivergent person in the workplace is that the concept itself is very nascent in Singapore … But when you tell somebody I’m autistic or I’m neurodivergent, even the bosses themselves, when well-intentioned, are like, okay, so, I know you need help, but like, how do you want me to help you? And so the problem is, how do you tell them what help you need in a way that would make them understand that, that I’m not taking anything for granted.”.

Along with communication support needs, participants also described feeling anxious, doubting themselves, and seeing their challenges as personal shortcomings. For example, when describing difficulties with interpreting sarcasm and navigating social interactions, participants sometimes framed these experiences through the language of personal lack (“just lacking in social skills, lacking in knowledge of social norms lah”; Freddy). This suggests that workplace challenges can become internalized as individual inadequacy rather than understood as a mismatch between interactional expectations and one’s natural way of processing information. Across these accounts, it becomes evident that support should help individuals identify what they need and communicate those needs in ways that feel comfortable and respectful. They should also encourage more positive ways of understanding social difficulties, reducing self-blame and anxiety about future interactions.

#### 3.2.2 Need for work design and tools that align with neurodivergent working styles

Participants noted that their success at work was often closely tied to how well their tasks suited their individual strengths. Roles that aligned with their cognitive preferences allow them to be more focused and successful in their work: “I do very badly at jobs where I need to do different things at the same time. Let’s say it’s a project. First, you got to write an email to someone, then you got to edit the work. After that, you got to talk to somebody else. And I really struggle with that. So for this job, I was only able to succeed because the job scope is very focused” (Ning). Environmental factors also played a crucial role in supporting job performance. Quiet spaces and control over stimuli were perceived as essential for concentration and well-being, with Rachel recounting their experience working in food and beverage service in a shopping mall: “It’s very noisy and very bright lighting, and it’s very overwhelming for me. So sometimes, like I don’t feel well in the middle and like, unable to complete the job.”.

Participants discussed LLMs as a potential neurodiversity-informed tool that could improve person-job fit by adapting how work demands are presented and supported. Specifically, they imagined AI features that could reduce mismatch between role demands and their cognitive processing styles by transforming complex or fragmented tasks into formats that are easier to act on, and by delivering information in ways aligned with individual preferences. For example, participants emphasized LLM outputs that are brief, jargon-free, and accompanied by adaptable visual formats that could support readability and support focus.

At the same time, participants stressed that such potential would depend on whether LLMs could be designed to align with autistic ways of thinking and working. Some felt that current tools remain limited in this respect. Lyon, for instance, suggested that AI should accommodate non-linear ways of organizing information rather than assuming a linear decision pathway. They described their thinking as “some sort of gigantic web where the details are always spun out” than “a straight line” and noted that current LLMs may not readily reflect or support this mode of sense-making. Others highlighted that effective support requires the system to recognize the user’s neurodivergent context and tailor responses accordingly. Ben noted that current tools often lack “situational awareness” of neurodivergence, while Nathan emphasized the need for user profiling so the system can adapt to individual differences and retain these preferences over time.

Taken together, these suggestions position LLMs not as a generic productivity aid, but as a potentially valuable part of neurodiversity-informed work design, if designed in ways that are responsive to autistic people’s cognitive styles, preferences, and support needs. This may hence help make work demands more adaptable to the person and, in turn, strengthen the person-job fit.

#### 3.2.3 Need for empowered access to support and accommodations

Even when participants knew what they need to be successful at work, they described multiple barriers to accessing those supports. Across accounts, these barriers clustered into two areas: first, gatekeeping and legitimacy requirements that shaped whether support could be requested safely and taken seriously; and second, practical access barriers related to help seeking and advocacy.

Participants described how support was often difficult to negotiate without disclosure, particularly in relation to work environment aspects that were controlled by supervisors. In these accounts, the ability to access adjustments was shaped by perceived negotiating power and whether one’s support needs had been formally recognized. As Ben shared: “The environment definitely is a significant factor in terms of being able to cope at the workplace. But often it’s not within your control, because the supervisor will just say, ‘Oh, this is the only desk we have left, ’ or, ‘This is your assigned seating arrangement, ’ so you might not have negotiating power over your supervisor to negotiate for a place, especially if you didn’t declare your support.”.

At the same time, participants described disclosure as risky, expressing concerns about negative judgements from supervisors, colleagues, and even clients. These concerns reflected fears of being perceived as unreliable, unsuitable, or burdensome, and that disclosure could alter workplace dynamics in ways that felt stigmatizing rather than supportive. “I want people to rely on me and for them to not see me as a liability, that kind of stuff. And then they might also walk on eggshells around me … I don’t want people to … sugarcoat stuff … So like, I guess it’s better if I don’t and I’ll just try to make do with what I have” (Nathan). Ben noted that even if they did disclose, limited awareness about neurodivergence could lead to misinterpretation of their needs. Uncomfortable, invalidating responses from co-workers (Ben: “Say things like, ‘You don’t look autistic”) made it harder to seek judgment-free support. Thus, some participants imagined LLM tools as collaborative thinking partners that they could turn to instead for guidance and problem-solving in a low-risk, judgment-free environment. For instance, Tim described using an LLM tool as an interactive sounding board: “Let’s say I ask the LLM, ‘I’m about to handle this project with many, many team members, ’ and they give me a general list of what to expect, what I can do. Then I can slowly feedback more of the nuances [and it can] adjust to my situation. Then it can give me a few different solutions. I can pick one, and then me and the LLM can discuss further”.

Beyond disclosure, participants described barriers at the level of day-to-day help seeking, including uncertainty about how to initiate communication with superiors, when it was appropriate to approach them, and what to raise. This reflects a “starting point” problem, where the pathway to support is not explicit and requires ongoing social judgement. Some participants also pointed to the value of an objective ally, such as a professional who could explain autism and support needs in a way that reduces misunderstanding and shifts advocacy away from the individual having to justify themselves repeatedly: “It actually helps to defuse a lot of the misunderstanding and tension already when you have a professional coming in to share objectively about what autism is. That allows the workplace to also manage their expectations: not lower, but manage their expectations of you” (Ben).

Building on the idea of credible allies, participants also envisioned LLMs as a tool that could function as a neutral guide within organizational structures, especially when traditional help-seeking pathways are limited or inaccessible. Oliver highlighted the potential for LLMs to support the navigation of organizational procedures, such as reporting complaints or understanding company policies: “If the AI tool can access the internet and scrape the company website for certain company policies that you might want to be aware of, say you have a formal complaint to make, or you face workplace discrimination. You want to figure out who to talk to about that specific thing, and you’re not comfortable talking to your direct supervisor. The AI can and should help you understand what the company procedures are.”.

Together, participants’ experiences suggest that access to support depends on clear and legitimate ways to ask for help, including navigating workplace hierarchies, having trusted allies, and feeling safe to disclose without fear of judgment or negative consequences. When these conditions were absent, support was experienced as discretionary and risky, which could then reduce the likelihood that participants would obtain the support they needed. As Deborah shared: “My psychiatrist wanna talk to my boss, but I also say no to it. Because later they say, ‘Because you got ASD. Then you are not suitable to be in this job.’”.

#### 3.2.4 Need for shared responsibility for workplace integration

Participants’ accounts suggested a need for shared responsibility for workplace integration, such that successful participation is not contingent on autistic employees alone to adapt to neurotypical norms. When integration was experienced as an individual responsibility, participants described pressures to mask, blend in, and minimize needs to preserve social cohesion. Henry reflected on the psychological toll of this approach, linking this sustained masking to burnout: “I do realize that it’s also possible for me to blend in with the neurotypicals as well as join in their activities. Typically that’s when burnouts are rather common”.

Others described downplaying their support needs to avoid burdening colleagues, reflecting a desire to maintain harmony even when it required self-suppression: “I don’t want to inconvenience them … I try to make do with what I have ‘cause like, I don’t wanna be seen as too overbearing” (Patrick). While such strategies may reduce interpersonal friction in the short term, they also point to a personal cost when integration depends on masking and suppression.

Participants also described shared responsibility as an interactional need, emphasizing reciprocity in communication rather than expecting only one person to adapt. Ben drew on the double empathy problem (55) to highlight that misunderstandings arise from mutual difficulties in perspective taking, not solely perceived autistic deficits. Lyon reflected on the unfairness of expecting autistic employees to adapt to these differences, noting that “most of them would still feel offended if we didn’t at least try. But on the other hand, it’s strictly unfair for everybody to expect us to do literally all the effort. That’s an unfair expectation.” These accounts suggest a need for workplace norms where colleagues and supervisors also take active responsibility for mutual understanding.

In terms of support, some participants envisioned the use of LLMs as a real-time mediator that could help distribute responsibility for communication more evenly. Gary described an imagined functionality in which the LLM observes multi-party interactions, detects potential miscommunications, and provides neutral summaries: “There’s a central AI that can listen to communication in meetings. Then if there is any miscommunication or whatsoever, the GenAI will automatically voice out and try to solve certain things or raise points missed, miscommunication, certain issues. Like sometimes [when] I say something, the other person says something, and we don’t understand each other, the AI will be like, ‘This is not what this person means.’” Patrick stressed that the LLM should attend to the external context and social environment to act as a bridge between the user and others: “It shouldn’t really mirror me? It should mirror the environment around me … A bridge between me and those around me.”.

Finally, participants pointed to structural and cultural conditions that can help share the responsibility for inclusion more fairly. In everyday social practices, as indicated in section 3.1.2, opting out of lunch conversations or social rituals could be misread as being “strange, ” “weird, ” or “antisocial, ” (Josephine) indicating the need for consent-based norms and legitimate opt out pathways that have no social penalties for choosing what works best for them. At a broader level, Ning described how working with colleagues who were “brought up in other countries which are more open to neurodivergence” made it feel “quite safe to disclose, ” suggesting that the extent to which shared responsibility is enacted in practice is often shaped by the norms and awareness embedded in workplace culture. In contrast, Singaporean workplaces were described as having nascent awareness and understanding of neurodivergence, which could make disclosure feel unsafe or trigger assumptions about incapability: “But I feel that Singaporeans also lack some awareness about [neurodivergence]. And also a bit judgmental. For example, if you don’t do certain things, then they may say [you’re] lazy … I feel that there isn’t this awareness that this is a real condition” (Ning).

Participants also described structural arrangements that reduced social disadvantage, such as remote working options where text-mediated communication was the norm, creating a more “level playing field, ” because social relationship demands were less central and not expected of them: “What I’m not actually able to do, they [colleagues] are not even doing it already because of how the current working environment [is]” (Alex). Together, these accounts suggest that workplace integration depends on shared responsibility, combining reciprocal communication practices with workplace structures that make participation flexible and safe to opt out of.

## 4 Discussion

Disability scholars have addressed emerging consequences of AI use for disability inclusion in Asian contexts (56, 57) but have not specifically addressed neurodivergent needs. Meanwhile, research on autism in Singapore has addressed structural conditions affecting autistic empowerment. These include gaps in institutional and social support (58), challenges in educational settings (59), and workplace challenges (31). However, there is a lack of research in Singapore addressing how autistic stakeholders experience employment, as well as how they imagine meeting their workplace needs in relation to possible support from LLM technologies, which is what this study aimed to address.

### 4.1 Challenging assumed neurotypicality of the workplace

Our first theme aligns with existing research documenting that autistic and neurodivergent individuals often face expectations to understand unstated social rules, workplace etiquette, and manage vague or ambiguous instructions (60, 61). Participants reported that instructions were often unclear, requiring them to infer supervisors’ expectations, while hierarchical norms and complex chain-of-command procedures made help-seeking feel risky or inaccessible. In response, participants emphasized the need for more explicit instructions, structured feedback, and approachable pathways for clarification. These findings corroborate prior research identifying direct communication and structured guidance as key enablers of workplace participation for autistic individuals (62).

Besides task ambiguity, participants’ accounts suggest that many workplaces place substantial, and often implicit, demands on executive functioning. In this sense, executive functioning can be understood as part of the hidden curriculum of work, namely the unspoken expectation that employees will independently plan, organize, prioritize, shift between tasks, and adapt flexibly as situations change (63), all of which also needs to be completed in a highly efficient manner. Rather than framing this as an individual deficit, our findings highlight a mismatch between workplace designs that routinely test these top-down processes and the limited scaffolding available when such demands are high or rapidly shifting. This mismatch may be rather consequential for autistic employees, given that executive functioning profiles in autistic adults do include challenges in domains such as working memory and cognitive flexibility (64). Research has shown that attention-deficit hyperactivity disorder (ADHD) often co-occurs in autistic adults, with population-based estimates indicating elevated rates relative to the general population. In our sample, approximately 20% of participants reported a co-occurring ADHD diagnosis, suggesting that executive functioning-related barriers may be salient for a substantial subgroup. Yet, supports that reduce or scaffold executive functioning demands, such as clearer prioritization, explicit task sequencing, and structured transition supports, were not often available or offered. In such cases, workers may be perceived as incompetent or not knowing how to do their job well, when these difficulties instead reflect a mismatch between job demands and how they work.

These experiences can also be understood through the Job Demands-Resources (JD-R) model, which explains workplace stress as arising from the imbalance between job demands and available resources (65). In this context, job demands (e.g., executive functioning demands, emotionally demanding social interactions, role ambiguity, unclear instructions) and resources (e.g., supervisory support, clear help-seeking pathways, explicit guidance) interact to shape employee well-being. When resources are limited, emerging evidence suggests that such imbalances may disproportionately impact autistic employees, with job demands more readily depleting resources, increasing emotional labor and stress (66). This in turn can give rise to autistic burnout, a phenomenon characterized by Raymaker et al. (67) as a state of incapacitation, exhaustion, and distress in every area of life, arising from chronic stress and mismatched expectations without adequate support (68). Exhaustion can then in turn negatively impact work engagement and job performance (62).

Social participation is another workplace domain where neurotypicality is often assumed. What counts as social participation and belonging varied across our participants. Several participants expressed discomfort with informal workplace activities they perceived as peripheral to performance. Consistent with prior research, autistic employees may attribute less social significance to workplace rituals, such as shared lunches or birthday celebrations, than their non-autistic peers (69, 70). When they opt out, it may reflect differing interpretations of the purpose and expectations of such practices, rather than disengagement (71).

Our analysis also points to fundamental differences in how communication and interactions are assumed in professional settings. In predominantly neurotypical workplaces, autistic employees are often expected to navigate these implicit norms on their own, which can create recurring strain. This dynamic is captured by the double empathy problem, which conceptualizes misunderstandings as arising from differences in how autistic and non-autistic individuals make sense of social cues and expectations (55). Within cross-neurotype interactions, both parties may find it challenging to accurately infer one another’s thoughts, feelings, and intentions (72). In other words, workplace strain arises from a mutual mismatch in communication styles and assumptions from both parties.

These experiences may be exacerbated by the Singapore sociocultural context. As an Asian society, Singapore is often characterized by a strong orientation toward sameness and conformity, with individuals being pressured to fit in rather than stand out (59). These sentiments are reflective of attitudes in other collectivistic cultures, where researchers have reported higher stigma against people who do not fit the norm and lower tolerance for diversity (42, 73). Such a sociocultural mindset places additional pressure on neurodivergent employees to fit in with a workplace, peers, and society that are largely neurotypical. Additionally, collectivistic norms may operate not only through expectations that autistic people conform, but also through pressures on co-workers to align with dominant group behavior, even when this takes exclusionary forms such as bullying. One participant, for instance, described being singled out by colleagues in ways that appeared to lessen once the main instigator left the workplace.

The expectation that all employees conform to neurotypical norms can constrain workplace inclusion, framing supports as “special” rather than as good work design. Without downplaying the very real ways autistic employees can struggle with workplace demands, some challenges described by participants (e.g., managing unclear expectations, navigating hierarchical communication) mirror some of the common hindrance stressors experienced by employees more broadly, including role ambiguity, job scope, and organizational politics (74), suggesting that many workers can benefit from supports in these areas. Adopting the neurodiversity lens reinforces this point by recognizing that there is no single “typical” brain profile (75); employees inevitably vary in strengths, processing styles, and support needs. From a support needs lens, the key distinction is therefore not who needs support versus who does not, but the degree and conditions under which support is required (76). For autistic employees, particular support is essential for consistent performance and well-being, while for others the same support may only be needed in certain contexts to reduce strain and support effective working. Recognizing neurodiversity and universalizing supports can thus strengthen both equity and workplace functioning, and it can also reduce the extent to which access depends on formal diagnosis or disclosure, given known barriers that can make diagnosis difficult to obtain and disclosure difficult to navigate. Designing workplaces around explicit communication, structured onboarding, clear prioritization, and predictable processes can therefore broaden access to support, reduce reliance on implicit norms, and create conditions where varied ways of working are anticipated rather than penalized.

### 4.2 Inclusivity through individual support and systemic change

Our second theme reflects participants’ views that creating inclusive workplaces requires the simultaneous support of individual employees and the enforcement of systemic processes that encourage and facilitate inclusive working practices. This aligns with prior research emphasizing that effective inclusion depends on a combination of individualized support and systemic practices that normalize and facilitate neurodivergent ways of working (18, 77).

A latent insight from our findings is that some participants perceived gaps in their own self-awareness and often framed these as deficits in relation to non-autistic peers. They imagined LLM tools functioning as a reflective aid, surfacing blind spots in their own conceptualizations of their strengths and support needs. Since these experiences of internalized social challenges have been linked in the literature to reduced work performance and well-being (18), our findings highlight the importance of supporting self-awareness and creating structures that mitigate the negative impacts of such challenges. Historically, autistic self-awareness has been approached from a deficit lens, often portraying autistic people as lacking insight into internal states and having an impaired sense of self (78). Such portrayals risk dehumanizing autistic individuals by overlooking their capacity for introspection. More recent work challenges this perspective, highlighting the empowering potential of self-recognition (79). In this way, the desire for tools that support reflection may surface a need for structured opportunities to make sense of oneself within environments that privilege neurotypical norms. Because effective accommodations depend on clear articulation of needs, supporting self-awareness and addressing internalized deficit narratives become crucial components of workplace empowerment.

However, beyond identifying their personal strengths and support requirements, autistic employees must also navigate whether these needs are considered legitimate by colleagues and supervisors. Disclosure can be a strategy to access accommodations, but it also carries the risk of scrutiny and further marginalization (80). Similar to the process of moral evaluation described by Dodier (81), where workers taking sick leave are assigned a moral position that indexes the perceived validity of their absence, autistic employees requesting accommodations may be implicitly judged on the perceived reasonableness of their needs. The heterogeneity of autism can exacerbate this issue. As autistic employees’ support needs and preferences vary, disclosure may be met with skepticism, with colleagues or supervisors questioning why one person requires certain accommodations while another does not. This potential for scrutiny may discourage disclosure, leading employees to instead rely on camouflaging (i.e., hiding autistic traits and attempting to act neurotypically) which has been identified as a risk factor for depression (82) and contributes to depletion of coping resources, increasing stress and burnout (83). As such, beyond the articulation of one’s needs, workplace inclusion depends on the systemic acceptance and structural support that acknowledge and normalize neurodivergent ways of working. Recent research indicates that current resolutions to workplace-based social challenges faced by autistic employees tend to be targeted toward the autistic individual rather than the workplace environment (e.g., the employee learning to adjust to the work culture on their own, 18). Systems-level acceptance is thus additionally important to avoid placing the sole responsibility of job fit on autistic individuals.

Yet, as neurodiversity perspectives gradually gain traction in Asian societies, autism advocacy and support must also consider socio-cultural and linguistic differences that affect how autistic people can seek empowerment (2). In Singapore’s context, researchers have found that autism is largely still seen as a disorder to be cured rather than differences to be affirmed, and autistic people feel that employers in Singapore are generally reluctant to accommodate their needs (58). This may be reflective of the wider sociocultural sentiment of conforming to social norms as noted above and reflects a need for greater neurodiversity awareness and acceptance on a societal level. These unique conditions affect how autistic people imagine localized constraints and possibilities for empowerment. Thus, in developing inclusive workplaces, it is imperative that culturally appropriate support is provided in tandem with systems-level acceptance to enable person-environment fit.

### 4.3 Contextualizing the potential for AI support

Participants’ accounts for desired affordances suggest that AI and LLM tools have the potential to address some of their workplace needs, thereby supporting a more effective and positive work experience. The affordances described by participants were primarily oriented towards supporting the autistic individual directly. For example, participants shared how they would like for LLMs to scaffold executive functioning via work decomposition, prioritizing competing demands, formulating and achieving stepwise plans, and preparing for transitions between routines or contexts, thereby reducing cognitive demands (84, 85). They would also like LLMs to facilitate social communication by helping users draft or rehearse messages, clarify requests, and generate contextually appropriate responses across different tones or levels of urgency (86).

Participants also expressed interest in LLM tools being used in group support contexts, although this was raised much less frequently. This may be because LLM tools are most introduced and encountered through one-to-one conversational interfaces such as ChatGPT (87). Participants shared how they would like for AI-mediated communication tools to facilitate mutual understanding between autistic and non-autistic colleagues, offering bidirectional support that helps both parties interpret each other’s communication styles (88). Such support is particularly valuable given that workplace frictions often stem from mismatched expectations or differing interpretations between autistic and non-autistic individuals. Education is widely recognized as crucial and efficacious in promoting awareness of neurodiversity, equipping non-autistic individuals with knowledge to support inclusive interactions (89). By providing neutral, evidence-based guidance, AI tools could lower barriers to shared responsibility as opposed to defaulting to ambivalence or avoidance of the autistic employee (90). Importantly, this approach preserves autistic agency, allowing autistic employees to engage authentically while validating their identity, compared to positioning them as needing to conform to neurotypical norms.

Beyond its potential as a workplace support, AI must also be examined through a broader lens that considers its sociocultural meanings, ethical tensions, and structural implications. Examining developments in AI for disability inclusion across Asian cities like Tokyo, Seoul, and Singapore, Goggin and Zhuang (56) argue that “disability is an important component of AI imaginaries in Asia, in that AI imaginaries draw on Asian disability identities and representations but also shape them” (p. 271). With AI being imagined as a revolutionary technology, it is inevitable that the consequences of mass adoption of AI will intimately affect the lives of autistic individuals, within and beyond the workplace. AI reproduces marginalization against neurodivergent and other disabled people through algorithmic biases that fail to reflect disabled lived experiences (57). It is therefore very urgent and important for scholars of neurodiversity and AI designers to engage each other in honest, sometimes difficult, dialogue. Within this dialogue, it is imperative that we also consider the implications of sociocultural norms surrounding disability, neurodivergence or neurodiversity, and AI technologies.

Culturally, there are also language considerations. AI tools have been primarily trained in English, so it is unsurprising that they are widely available in English, though other languages are increasingly being supported (91). Some participants in our study similarly noted that AI tools need to be attuned to local linguistic and cultural contexts (such as the use of Singlish in many Singaporean workplaces) to be effective. However, at present, LLMs exhibit a broad range of capabilities in this regard, where some may be able to communicate with users in Asian languages (including Singlish) but are incapable of responding with mixed-language outputs (92). Such contextual considerations must be addressed through concerted efforts to include local neurodivergent communities in processes for co-designing AI solutions. The present study constitutes a modest, exploratory attempt to do so in the Singapore context, thereby addressing the lack of research integrating AI and autistic needs in Singapore.

One difficult tension to address is whether AI tools for autistic people will reinforce their marginalization by playing into social expectations for neurodivergent people to conform to neurotypical standards. While AI tools can help autistic workers to be more productive or communicate better at work, there is a genuine risk that this could increase workplace expectations that the onus is on autistic individuals to use AI tools to “cope” better, rather than on neurotypical society to accommodate their differences, as already evidenced in the literature on AI interventions and autism (43). Ultimately, it must be emphasized that AI tools are necessarily limited in their ability to address autistic needs. Neurodivergent needs are diverse, complex, and can be highly personal and circumstantial (77). While technological solutions are often a necessary band-aid in doing all that we can to address existing inequalities, changing social structures (including norms, policies, institutions, and worldviews) is core to addressing neurodivergent needs in a world designed and structured to disable and disadvantage neurodivergent people. It is thus crucial that tools aimed at supporting neurodivergent individuals, whether AI tools or otherwise, are affirming rather than pathologizing of neurodivergence, and implemented together with measures that target systemic inclusivity. In our companion paper, we take an initial step towards discussing this issue by examining participants’ interactions and chatbot responses with a chatbot prototype designed to reflect autism-affirming principles.

### 4.4 Limitations and future directions

While this study represents one of the first qualitative explorations of autistic adults’ perspectives on workplace inclusion in Singapore, it is important to interpret the findings within their specific context. First, participants were recruited from a single country. While the findings may not be necessarily generalizable across countries with different workplace cultures, conducting the study in an underrepresented geographical and sociocultural context such as Singapore allows us to contribute new insights into workplace experiences that are rarely documented in the current body of autism literature.

Second, our findings may primarily reflect shorter term work experiences and may not fully capture the challenges associated with longer-term workplace integration, as 65% of our participants had less than 4 years of working experience. Participants were primarily employed in office or retail environments, which may limit the generalizability of our findings to other industries. Despite open recruitment efforts, participants were largely from one ethnic background (Chinese), which may reflect Singapore’s demographic profile but could limit the transferability of findings to autistic individuals from minority ethnic backgrounds locally. This consideration is important given emerging local evidence of ethnic-group differences in autism-related pathways and outcomes (93), alongside evidence from the mental health literature that stigma and help-seeking may vary across ethnic groups (94), which may also then impact their experiences in the workplace context.

Another limitation of this study is that our recruitment and group discussion design likely favored autistic adults who were comfortable participating in spoken small-group discussions and who had a strong command of expressive English. As a result, our sample likely did not capture the full range of communication preferences represented within the autistic community (95) and may underrepresent autistic adults who rely on alternative communication modalities (e.g., using augmentative and alternative communication (AAC) devices, written communication, or asynchronous communication) or who prefer one-to-one settings. The findings should therefore be interpreted as reflecting the experiences of autistic adults who can and are willing to engage in real-time, verbally mediated group discussion, rather than the broader autistic adult population. This sampling constraint may also shape which needs were most salient, potentially amplifying themes related to rapid conversational demands and turn-taking while underrepresenting support needs that arise in other communication contexts (e.g. barriers to AAC use at the workplace; 96). Future work should incorporate complementary methods and participation options (e.g., one-to-one interviews, written or asynchronous inputs, and AAC-accessible formats) to better capture the diversity of communication profiles and participation preferences among autistic adults.

Nonetheless, this study strived to incorporate several methodological strengths in its framework. A participatory approach engaged both autistic and non-autistic researchers in design, data collection, analysis, and write-up, supporting inclusive research practices. Our usage of RTA allowed data-driven generation of themes, keeping participants’ voices central while acknowledging researcher influence.

Future work could explore perspectives of autistic adults across a wider range of employment contexts and industries to capture the diversity and intersectional influences of workplace experiences. Broadening recruitment of participants from a wider range of socioeconomic and ethnic backgrounds could also further reveal how intersecting factors influence access to accommodations, choices around disclosure, and professional development. Future research could incorporate perspectives from stakeholders such as employers, colleagues, or job coaches, to provide a more holistic understanding of workplace dynamics from multiple vantage points. As this study serves as an initial step towards identifying autistic workplace needs, future work could expand this line of inquiry by collecting larger-scale survey data to identify and prioritize key workplace support needs among autistic adults, thereby informing the targeted and timely development of supports for improved employment experiences.

## 5 Conclusion

This study sought to initiate dialogue on workplace inclusion in Singapore by foregrounding autistic employees’ experiences. This analysis revealed key barriers that impede workplace success for autistic adults, including navigating ambiguous instructions, managing executive functioning related task demands, and negotiating social interactions shaped by neurotypical norms. However, our findings suggest that workplace realities are shaped by broader systemic factors, with persistent stigma being one of the most enduring barriers to inclusion. Importantly, the responsibility for overcoming these challenges should not rest solely on the employee; systemic change is essential to truly foster inclusive workplaces. Positioned at the helm of workplace support, AI heralds the potential to transform inclusion practices. Thoughtfully designed, user-centered AI tools can support autistic employees while respecting neurodivergent ways of working, helping to redistribute the burden of adaptation and facilitate equitable workforce participation.

## Data Availability

The datasets presented in this article are not readily available because not all participants consented to data sharing. Requests to access the datasets should be directed to Delia Kan, delia.kan@nie.edu.sg.
